# Lack of Rhes Increases MDMA-Induced Neuroinflammation and Dopamine Neuron Degeneration: Role of Gender and Age

**DOI:** 10.3390/ijms20071556

**Published:** 2019-03-28

**Authors:** Giulia Costa, Pier Francesca Porceddu, Marcello Serra, Maria Antonietta Casu, Valentina Schiano, Francesco Napolitano, Annalisa Pinna, Alessandro Usiello, Micaela Morelli

**Affiliations:** 1 Department of Biomedical Sciences, Section of Neuroscience, University of Cagliari, Cittadella Universitaria, 09042 Monserrato, Italy; giulia.costa83@gmail.com (G.C.); fra8714@hotmail.it (P.F.P.); marcelloserra92@gmail.com (M.S.); morelli@unica.it (M.M.); 2 Institute of Translational Pharmacology, UOS of Cagliari, National Research Council of Italy, Scientific and Technological Park of Sardinia POLARIS, 09010 Pula, Italy; mariaantonietta.casu@ift.cnr.it; 3 Laboratory of Behavioral Neuroscience, Ceinge Biotecnologie Avanzate, 80145 Naples, Italy; schiano@ceinge.unina.it (V.S.); napolitano@ceinge.unina.it (F.N.); usiello@ceinge.unina.it (A.U.); 4 Department of Molecular Medicine and Medical Biotechnology, University of Naples “Federico II”, 80131 Naples, Italy; 5 Neuroscience Institute, National Research Council of Italy, Cittadella Universitaria, 09042 Monserrato, Italy; 6 Department of Environmental, Biological and Pharmaceutical Sciences and Technologies, University of Campania, Luigi Vanvitelli, 81100 Caserta, Italy; 7 National Institute of Neuroscience (INN), University of Cagliari, 09100 Cagliari, Italy

**Keywords:** astrocytes, microglia, neurodegeneration, dopamine, MDMA, Rhes knockout mice, psychosis

## Abstract

Ras homolog enriched in striatum (Rhes) is a protein that exerts important physiological functions and modulates psychostimulant drug effects. On this basis, the object of this study was to assess 3,4-methylenedioxymethamphetamine (MDMA) effects on microglial (CD11b) and astroglial (GFAP) activation and on dopamine neuron degeneration (TH) in wild-type (WT) and Rhes knockout (KO) male and female mice of different ages. Motor activity was also evaluated. Adult (3 months) MDMA-treated mice displayed an increase in GFAP-positive cells in striatum (STR), whereas the substantia nigra pars compacta (SNc) was affected only in male mice. In these mice, the increase of CD11b was more extensive including STR, SNc, motor cortex (CTX), ventral tegmental area (VTA), and nucleus accumbens (NAc). MDMA administration also affected TH immunoreactivity in both STR and SNc of male but not female WT and Rhes KO mice. In middle-aged mice (12 months), MDMA administration further increased GFAP and CD11b and decreased TH immunoreactivity in STR and SNc of all mice. Finally, MDMA induced a higher increase of motor activity in adult Rhes KO male, but not female mice. The results show that Rhes protein plays an important role on MDMA-mediated neuroinflammation and neurodegeneration dependent on gender and age, and confirm the important role of Rhes protein in neuroinflammatory and neurodegenerative processes.

## 1. Introduction

Ras homolog enriched in striatum (Rhes) belongs to the Ras superfamily that includes a group of small guanosine triphosphate (GTP)-binding proteins that exert pleiotropic effects on cell function [1]. A number of studies have demonstrated that Rhes knockout (KO) animals show alterations reminiscent of human psychiatric diseases, including deficits in prepulse inhibition of startle reflex and exacerbation of behavioral responses induced by amphetamine [2,3,4,5,6]. Moreover, a previous study of our laboratory demonstrated that Rhes KO male mice spontaneously develop an age-dependent loss of tyrosine hydroxylase (TH)-positive neurons in the substantia nigra pars compacta (SNc) and a progressive deficit in motor coordination and balance [7].

In addition to its striatum (STR) enrichment [2,3,4,5,6], Rhes mRNA is also localised in cortical and midbrain regions of rodents and primates [7,8], and modulates striatal dopamine (DA) and adenosine-related signal transduction and behaviours [2,3,4,5,9], further confirming its modulatory role in psychiatric and neurological diseases in which DA is involved.

In a recent study, our laboratory investigated the contribution of Rhes to neuroinflammation, demonstrating that lack of Rhes caused an increase of astrogliosis and microgliosis in KO mice [10]. These findings are particularly important since epidemiological and experimental evidence indicate that, among the possible etiopathogenic factors, neuroinflammation is one of major driver for progression of psychiatric disorders such as schizophrenia [11,12,13]. Moreover, glial cell activation is a key player in DAergic neuron degeneration, such as in Parkinson’s disease (PD) [14,15,16].

Among the amphetamine-related drugs, 3,4-methylenedioxymethamphetamine (MDMA, also known as “ecstasy”) is one of the most popular psychostimulants producing psychosis [17,18,19,20]. Moreover, preclinical data in mice have shown that MDMA decreases the levels of both DA and DA transporter in the STR [21,22,23,24,25,26,27,28,29] and reduces the immunoreactivity for TH in both STR and SNc [20,30]. Interestingly, the neurotoxic effects observed in the nigrostriatal system of mice treated with MDMA were paired with neuroinflammatory responses, and in particular with an increase in astrogliosis and microgliosis, as well as neuroinflammatory cytokines, including interleukin-1β (IL-1β) and tumour necrosis factor-α (TNF-α) [31,32]. Finally, MDMA-induced glial changes frequently paralleled a long-lasting and dose-dependent stimulation of motor activity [33,34].

On the basis of this knowledge, and considering that PD is more frequent in males than females, while both PD and schizophrenia are age dependent, we aimed to investigate the effect of the psychostimulant MDMA in inducing neuroinflammatory and neurotoxic effects in male and female Rhes KO mice at different ages. Neuroinflammation was evaluated by measuring microglial and astroglial activation through immunoreactivity for complement type 3 receptor (CD11b) and glial fibrillary acidic protein (GFAP), whereas neurodegeneration was assessed by means of TH immunoreactivity, in the motor cortex (CTX), STR, nucleus accumbens (NAc), SNc and VTA. Finally, consistent with the well-established role of MDMA treatment in enhancing locomotor activity in mice [35], as an index of its psychostimulant efficacy, we evaluated the potential contribution of Rhes to motor hyperactivity upon the administration of MDMA.

## 2. Results

### 2.1. Effects of MDMA on GFAP Immunoreactivity

#### 2.1.1. Adult Males and Females

**Figure 1:**
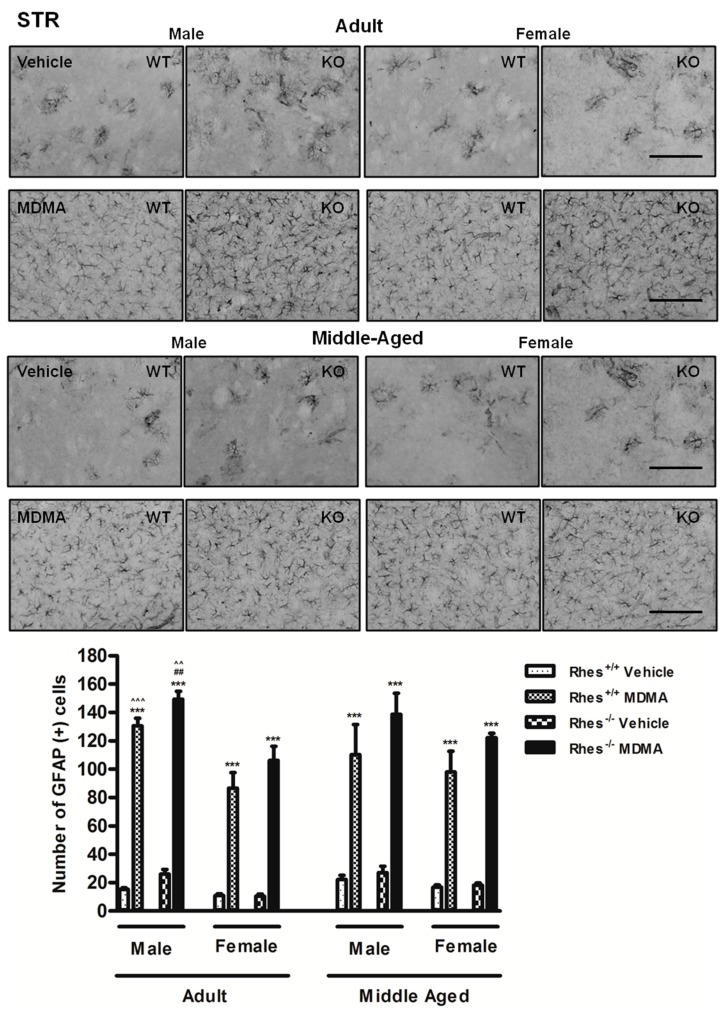
Representative sections (20× magnification) of the striatum (STR) immunostained for the glial fibrillary acidic protein (GFAP) of adult (upper photograms) and middle-aged (lower photograms) male and female wild-type (WT) and Rhes knockout (KO) mice treated with vehicle or MDMA (4 × 20 mg/kg i.p.; 2-h intervals). The histogram shows the number of GFAP-positive cells in the STR. Values are expressed as mean ± S.E.M. Number of mice per group: vehicle-treated WT adult male: *n* = 5 and female: *n* = 7; MDMA-treated WT adult male: *n* = 5 and female: *n* = 9; vehicle-treated WT middle-aged male: *n* = 11 and female: *n* = 6; MDMA-treated WT middle-aged male: *n* = 9 and female *n* = 6; vehicle-treated Rhes KO adult male: *n* = 5 and female: *n* = 9; MDMA-treated Rhes KO adult male: *n* = 4 and female: *n* = 10; vehicle-treated Rhes KO middle-aged male: *n* = 8 and female: *n* = 6; MDMA-treated Rhes KO middle-aged male: *n* = 6 and female: *n* = 5. *** *p* < 0.0005 compared with the respective vehicle; ^##^ *p* < 0.005 compared with the age- and gender-matched WT mice; ^^^^ *p* < 0.005 and ^^^^^ *p* < 0.0005 compared with the age- and genotype-matched female mice by Tukey post hoc test. Scale bar = 50 µm.

In adult mice, MDMA administration induced a significant increase in the number of GFAP-positive cells in STR in both male and female WT and Rhes KO mice, compared with the respective vehicle-treated mice (Figure 1).

In CTX and NAc, no significant increase in GFAP levels were observed, whereas MDMA administration to Rhes KO male mice induced a significant increase in GFAP-positive cells in VTA as compared with female mice (Table 1). For more details on the statistical analysis please see the Supplementary Materials.

#### 2.1.2. Middle-Aged Males and Females

**Figure 2:**
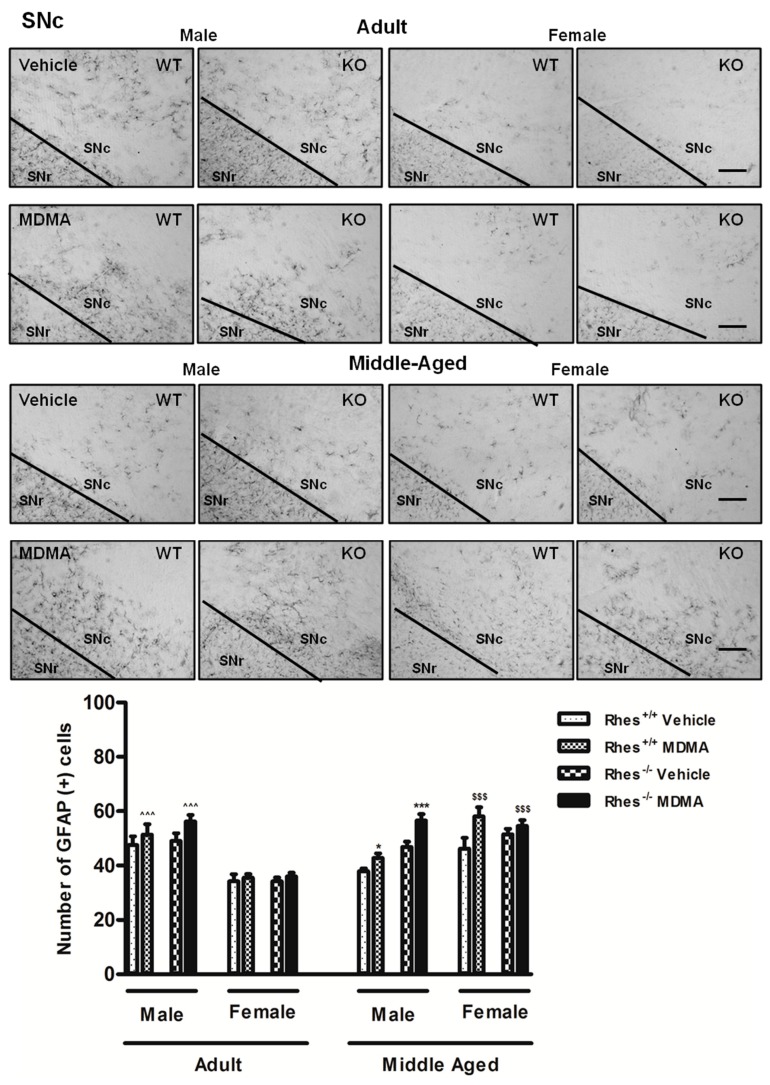
Representative sections (10× magnification) of the substantia nigra pars compacta (SNc) immunostained for the glial fibrillary acidic protein (GFAP) of adult (upper photograms) and middle-aged (lower photograms) male and female WT and Rhes KO mice treated with vehicle or MDMA (4 × 20 mg/kg i.p.; 2-h intervals). The histogram shows the number of GFAP-positive cells in the SNc. Values are expressed as mean ± S.E.M. Number of mice per group: vehicle-treated WT adult male: *n* = 5 and female: *n* = 8; MDMA-treated WT adult male: *n* = 5 and female: *n* = 9; vehicle-treated WT middle-aged male: *n* = 11 and female: *n* = 6; MDMA-treated WT middle-aged male: *n* = 8 and female: *n* = 6; vehicle-treated Rhes KO adult male: *n* = 5 and female: *n* = 9; MDMA-treated Rhes KO adult male: *n* = 5 and female: *n* = 10; vehicle-treated Rhes KO middle-aged male: *n* = 7 and female: *n* = 6; MDMA-treated Rhes KO middle-aged male: *n* = 8 and female: *n* = 5. * *p* < 0.05 and *** *p* < 0.0005 compared with the respective vehicle; ^^^^^ *p* < 0.0005 compared with the age- and genotype-matched female mice; ^$$$^ *p* < 0.0005 compared with the respective adult mice by Tukey post hoc test. Scale bar = 50 µm.

In middle-aged mice, MDMA administration induced a significant increase in the number of GFAP-positive cells in STR in both male and female WT and Rhes KO mice, compared with their respective vehicle-treated groups (Figure 1). In SNc, MDMA administration in male WT and Rhes KO mice induced a significant increase in the number of GFAP-positive cells compared with the respective vehicle-treated mice (Figure 2). Finally, the increase observed in SNc of female WT and Rhes KO mice was also significant as compared with adult mice (Figure 2). In CTX, NAc or VTA, no significant increase in GFAP levels were observed (Table 1). For more details on the statistical analysis please see the Supplementary Materials.

### 2.2. Effects of MDMA on CD11b Immunoreactivity

#### 2.2.1. Adult Males and Females

**Figure 3:**
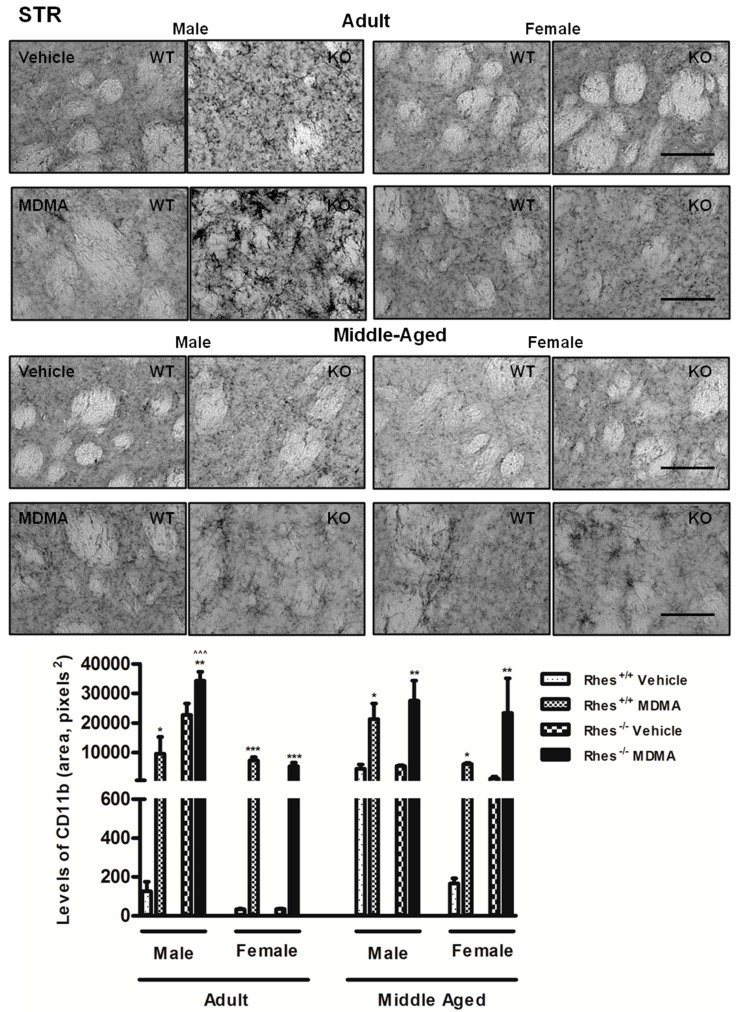
Representative sections (20× magnification) of the striatum (STR) immunostained for the complement type 3 receptor (CD11b) of adult (upper photograms) and middle-aged (lower photograms) male and female WT and Rhes KO mice treated with vehicle or MDMA (4 × 20 mg/kg i.p.; 2-h intervals). The histogram shows the area occupied by grey values above a threshold, calculated and expressed as square pixels in STR. Values are expressed as mean ± S.E.M. Number of mice per group: vehicle-treated WT adult male: *n* = 5 and female: *n* = 8; MDMA-treated WT adult male: *n* = 5 and female: *n* = 9; vehicle-treated WT middle-aged male: *n* = 9 and female: *n* = 6; MDMA-treated WT middle-aged male: *n* = 7 and female: *n* = 6; vehicle-treated Rhes KO adult male: *n* = 5 and female: *n* = 9; MDMA-treated Rhes KO adult male: *n* = 5 and female: *n* = 10; vehicle-treated Rhes KO middle-aged male: *n* = 6 and female: *n* = 6; MDMA-treated Rhes KO middle-aged male: *n* = 8 and female: *n* = 4. * *p* < 0.05, ** *p* < 0.005 and *** *p* < 0.0005 compared with the respective vehicle; ^^^^^ *p* < 0.0005 compared with the age- and genotype-matched female mice by Tukey post hoc test. Scale bar = 50 µm.

In adult mice, MDMA administration in male WT and Rhes KO mice induced a significant increase in the levels of CD11b in CTX, STR, NAc and VTA compared with the respective vehicle-treated groups (Figure 3 and Table 2).

**Figure 4:**
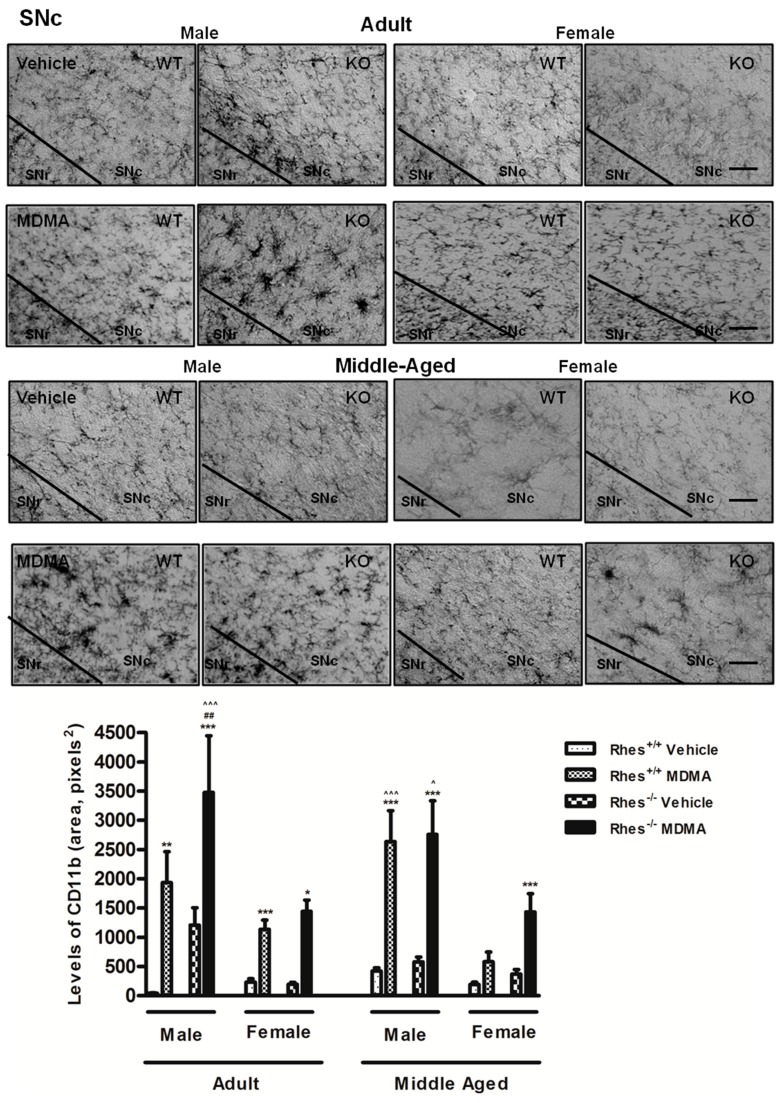
Representative sections (20× magnification) of the substantia nigra pars compacta (SNc) immunostained for the complement type 3 receptor (CD11b) of adult (upper photograms) and middle-aged (lower photograms) male and female WT and Rhes KO mice treated with vehicle or MDMA (4 × 20 mg/kg i.p.; 2-h intervals). The histogram shows the area occupied by grey values above a threshold, calculated and expressed as square pixels in SNc. Values are expressed as mean ± S.E.M. Number of mice per group: vehicle-treated WT adult male: *n* = 5 and female: *n* = 8; MDMA-treated WT adult male: *n* = 5 and female: *n* = 8; vehicle-treated WT middle-aged male: *n* = 10 and female: *n* = 6; MDMA-treated WT middle-aged male: *n* = 8 and female: *n* = 5; vehicle-treated Rhes KO adult male: *n* = 5 and female: *n* = 9; MDMA-treated Rhes KO adult male: *n* = 5 and female: *n* = 10; vehicle-treated Rhes KO middle-aged male: *n* = 6 and female: *n* = 6; MDMA-treated Rhes KO middle-aged male: *n* = 8 and female: *n* = 5. * *p* < 0.05, ** *p* < 0.005 and *** *p* < 0.0005 compared with the respective vehicle; ^##^ *p* < 0.005 compared with the age- and gender-matched WT mice; ^^^ *p* < 0.05, ^^^^^ *p* < 0.0005 compared with the age- and genotype-matched female mice by Tukey post hoc test. Scale bar = 50 µm.

Moreover, MDMA administration in female WT and Rhes KO mice induced a significant increase in the levels of CD11b in STR, although this increase was less marked as compared with the respective KO male mice (Figure 3). In SNc, MDMA administration in adult male and female WT and Rhes KO mice induced an increase in levels of CD11b compared with the respective vehicle-treated mice (Figure 4). 

Furthermore, the increase observed in SNc of male Rhes KO mice was significant as compared with WT and with KO female mice (Figure 4). For more details on the statistical analysis please see the Supplementary Materials.

#### 2.2.2. Middle-Aged Males and Females

In middle-aged mice, MDMA administration induced a significant increase in the levels of CD11b in CTX, STR, and VTA of male WT and Rhes KO mice, compared with the respective vehicle-treated mice (Figure 3 and Table 2), although in CTX this increase was less marked in female as compared with the respective male mice (Table 2). Moreover, MDMA administration in female WT and Rhes KO mice induced a significant increase in the levels of CD11b in STR and VTA, compared with the respective vehicle-treated mice (Figure 3 and Table 2). In SNc, MDMA administration in middle-aged male WT and Rhes KO mice and in female Rhes KO mice induced a significant increase in the levels of CD11b compared with the respective vehicle-treated mice (Figure 4). Finally, the increase observed in SNc of male WT and Rhes KO mice was also significant as compared with the respective female mice (Figure 4). For more details on the statistical analysis please see the Supplementary Materials.

Collectively, results from GFAP and CD11b immunoreactivity suggest that Rhes might be more involved in microglia-driven mechanisms rather than in astrocyte function. Moreover, CD11b and TH immunoreactivity suggest that the lack of Rhes protein affect in particular male mice especially with aging, but also makes middle-aged female mice more vulnerable to MDMA.

### 2.3. Effects of MDMA on TH Immunoreactivity

#### 2.3.1. Adult Males and Females

**Figure 5:**
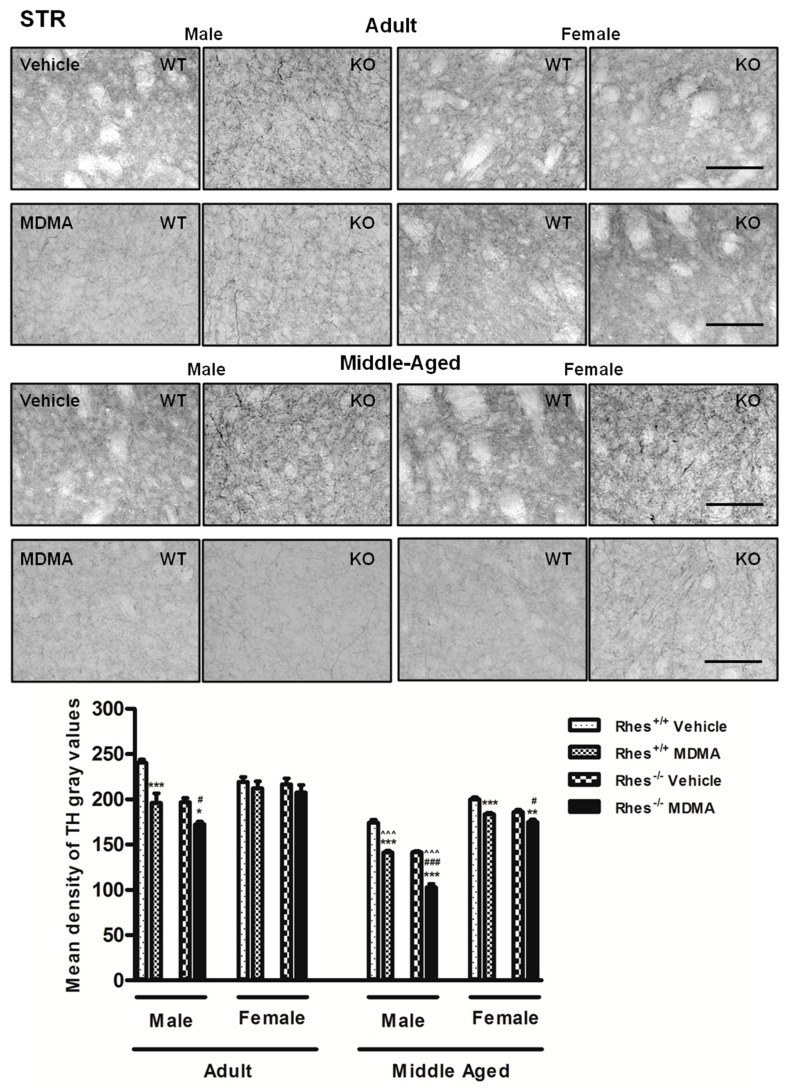
Representative sections (20× magnification) of the striatum (STR) immunostained for tyrosine hydroxylase (TH) of adult (upper photograms) and middle-aged (lower photograms) male and female WT and Rhes KO mice treated with vehicle or MDMA (4 × 20 mg/kg i.p.; 2-h intervals). The histogram shows the mean density of grey values of TH-positive fibres in the STR. Values are expressed as mean ± S.E.M. Number of mice per group: vehicle-treated WT adult male: *n* = 5 and female: *n* = 8; MDMA-treated WT adult male: *n* = 5 and female: *n* = 9; vehicle-treated WT middle-aged male: *n* = 9 and female: *n* = 6; MDMA-treated WT middle-aged male: *n* = 9 and female: *n* = 6; vehicle-treated Rhes KO adult male: *n* = 5 and female: *n* = 9; MDMA-treated Rhes KO adult male: *n* = 5 and female: *n* = 9; vehicle-treated Rhes KO middle-aged male: *n* = 8 and female: *n* = 6; MDMA-treated Rhes KO middle-aged male: *n* = 8 and female: *n* = 5. * *p* < 0.05, ** *p* < 0.005 and *** *p* < 0.0005 compared with the respective vehicle; ^#^ *p* < 0.05 and ^###^ *p* < 0.0005 compared with the age- and gender-matched WT mice; ^^^^^ *p* < 0.0005 compared with the age- and genotype-matched female mice by Tukey post hoc test. Scale bar = 50 µm.

In adult mice, MDMA administration to male WT and Rhes KO mice induced a significant decrease in the density of TH-positive fibres in STR compared with the respective vehicle, whereas adult female WT and Rhes KO mice were not affected (Figure 5).

**Figure 6:**
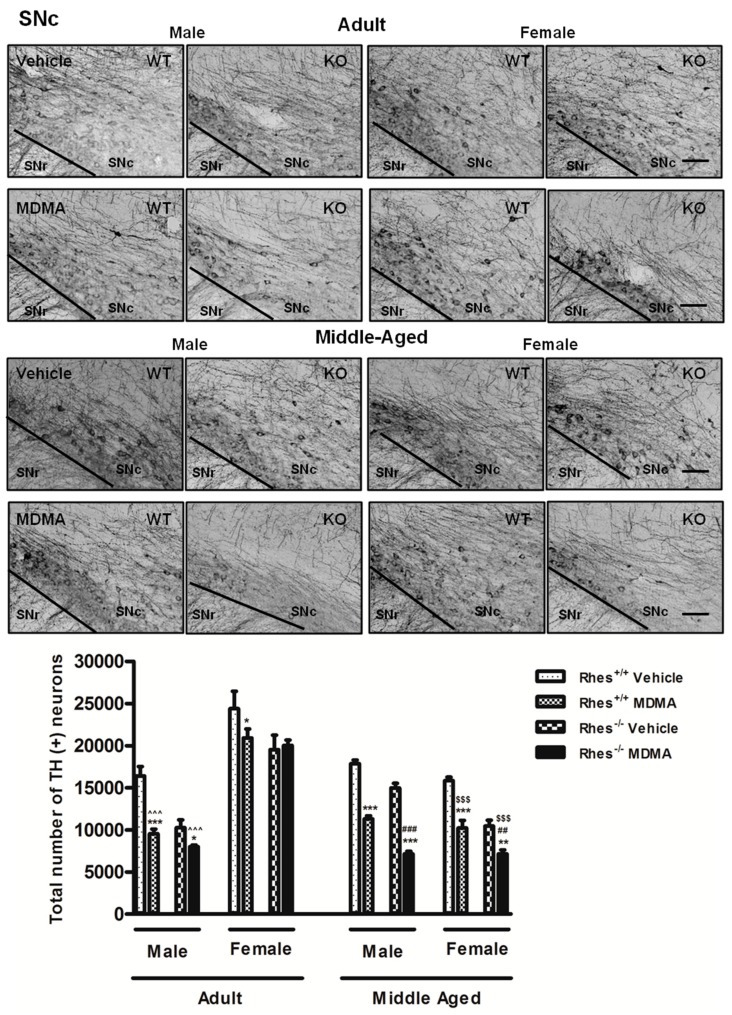
Representative sections (10× magnification) of the substantia nigra pars compacta (SNc) immunostained for tyrosine hydroxylase (TH) of adult (upper photograms) and middle-aged (lower photograms) male and female WT and Rhes KO mice treated with vehicle or MDMA (4 × 20 mg/kg i.p.; 2-h intervals). The histogram shows the number of TH-positive neurons in the SNc, calculated by stereological analysis in the SNc. Values are expressed as mean ± S.E.M. Number of mice per group: vehicle-treated WT adult male: *n* = 8 and female: *n* = 8; MDMA-treated WT adult male: *n* = 8 and female: *n* = 9; vehicle-treated WT middle-aged male: *n* = 12 and female: *n* = 6; MDMA-treated WT middle-aged male: *n* = 7 and female: *n* = 6; vehicle-treated Rhes KO adult male: *n* = 8 and female: *n* = 9; MDMA-treated Rhes KO adult male: *n* = 8 and female: *n* = 10; vehicle-treated Rhes KO middle-aged male: *n* = 8 and female: *n* = 6; MDMA-treated Rhes KO middle-aged male: *n* = 6 and female: *n* = 5. * *p* < 0.05, ** *p* < 0.005 and *** *p* < 0.0005 compared with the respective vehicle; ^##^ p <0.005 and ^###^ *p* < 0.0005 compared with the age- and gender-matched WT mice; ^^^^^ *p* < 0.0005 compared with the age- and genotype-matched female mice; ^$$$^ *p* < 0.0005 compared with the respective adult by Tukey post hoc test. Scale bar = 50 µm.

Moreover, the decrease observed in STR of male Rhes KO mice was also significant as compared with WT mice (Figure 5). In SNc, MDMA administration in adult male WT and Rhes KO and in female WT mice induced a significant decrease in the total number of TH-positive neurons compared with the respective vehicle-treated groups (Figure 6). For more details on the statistical analysis please see the Supplementary Materials. In CTX, NAc or VTA, no significant decrease in TH levels was observed (Table 3).

#### 2.3.2. Middle-Aged Males and Females

In middle-aged mice, MDMA administration induced a significant decrease in the density of TH-positive fibers in STR in both male and female WT and Rhes KO mice, compared with the respective vehicle-treated mice (Figure 5). Moreover, the decrease observed in STR of male Rhes KO mice was also significant as compared with WT mice and with the respective females (Figure 5). In SNc, MDMA administration in male and female WT and Rhes KO mice induced a significant decrease in the total number of TH-positive neurons compared with the respective vehicle (Figure 6). Moreover, the decrease observed in SNc of male and female Rhes KO mice was also significant as compared with WT mice (Figure 6). Finally, the decrease observed in SNc of female WT and Rhes KO mice was also significant as compared with the respective adult mice (Figure 6). In NAc and VTA, no significant decrease in TH levels were observed, whereas Rhes KO male mice showed a decrease in TH levels in CTX as compared with the respective female mice (Table 3). For more details on the statistical analysis please see the Supplementary Materials.

### 2.4. Motor Activity after MDMA in Adult Mice

**Figure 7:**
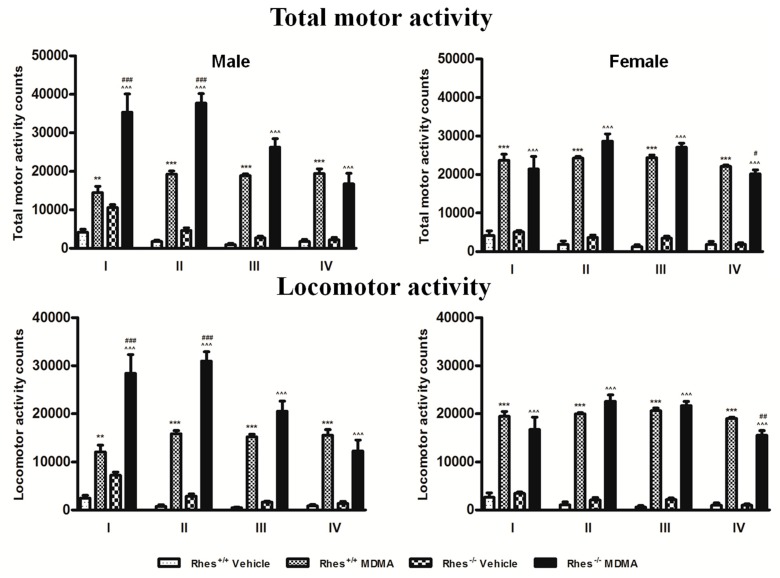
The histograms show the total motor (left panel) and locomotor (right panel) activity counts registered over the course of the treatment of adult mice, reported as total counts calculated for each MDMA administration. Activity counts were taken every 15 min for a total of four evaluations (cumulative time, 1 h) within each MDMA administration (I, II, III, IV). Mice were treated with vehicle (saline, male WT: *n* = 6 and Rhes KO: *n* = 6; female WT: *n* = 5 and Rhes KO: *n* = 5) or MDMA (4 × 20 mg/kg i.p.; male WT: *n* = 6 and Rhes KO: *n* = 5; female WT: *n* = 4 and Rhes KO: *n* = 5). Values are expressed as mean ± S.E.M. ** *p* < 0.005 and *** *p* < 0.0005 vs vehicle-treated WT mice; ^^^^^ *p* < 0.0005 vs vehicle-treated Rhes KO mice; ^#^ *p* < 0.05, ^##^ *p* < 0.005, ^###^ *p* < 0.0005 vs MDMA compared with the age- and gender-matched WT mice by Tukey post hoc test.

MDMA induced a significant increase in both locomotor and total motor activity (locomotor plus stereotypies) of adult WT and Rhes KO male and female mice as compared with the respective vehicle for from the first to the fourth administration (Figure 7).

Moreover, the locomotor and total motor activity of MDMA-treated Rhes KO male mice significantly increased compared with that of WT mice for the first two administrations (Figure 7), but this increase was not observed in female mice. Conversely, a significant decrease in both total motor activity and locomotor activity was observed in female Rhes KO mice as compared with WT mice after the fourth administration (Figure 7).

## 3. Discussion

The principal finding of this study is that Rhes protein influences MDMA-induced neuroinflammatory processes and DA neuron degeneration and does it in a gender and age dependent manner.

Starting from results of our research group showing that adult and middle-aged Rhes KO mice develop a spontaneous and age-dependent increase in astroglial and microglial cells, in both STR and SNc, which was more marked in male than in female mice [10], the present study examined the role of Rhes protein on the effects of the psychostimulant drug MDMA on GFAP and CD11b and evaluated if its effects were influenced by age and gender. Results show that glial activation in the nigrostriatal and mesocortical systems in response to MDMA administration is increased in Rhes KO adult male mice as compared with WT mice only in selective cases (GFAP in STR and CD11b in SNc), while decrease in TH in KO mice was observed in most cases. Moreover, while adult female KO mice do not show glial activation and decrease in TH as compared to WT, they become susceptible to DA neuron degeneration with age, suggesting a resistance in response of female as compared with male. Different preclinical reports suggest that amphetamine-related drugs may be one of the multiple factors leading to the neuroinflammation and DAergic neuron degeneration that, in turn, might cause psychiatric or neurological disorders in humans [11,12,13,25,28,36,37]. Moreover, preclinical results have demonstrated that MDMA, similarly to methamphetamine and other amphetamine-related drugs, induces neuroinflammation and toxicity to DAergic neurons, which is long lasting [30,34,38,39,40,41]. However, ad hoc studies on the occurrence of MDMA-induced neuroinflammation and neurotoxic damage in humans are limited by the fact that amphetamine-related drugs are illegal and produce psychotic-like behaviour [20]. For all these reasons, and for the role played by Rhes in DAergic neuron survival and in neuroinflammation described in the introduction, the objective of this research work was to study glial cell activation and DAergic neuron toxicity in mice lacking the Rhes protein with the aim of determining specific factors that may contribute to MDMA-induced noxious effects. The findings of this study acquire particular importance in view of the lack of study in humans and help the comprehension of the factors that are at the basis of neuroinflammation and neurodegeneration.

In relation to GFAP, the results obtained show that MDMA produced significantly higher activation of GFAP-positive cells in STR and SNc in male than female mice. Although an increase in the number of GFAP-positive cells was observed in SNc of female WT and Rhes KO with ageing, no differences were observed compared with the respective vehicle, whereas middle-aged male WT and Rhes KO mice displayed an increased number of GFAP-positive cells in the same area as compared with vehicle.

Regarding results on CD11b, immunoreactive cells were increased in both the nigrostriatal and mesocortical systems after MDMA and, again, a higher response was observed in adult males as compared with female mice.

The findings obtained in our study may be relevant for clarifying the role of Rhes in neuroinflammatory processes that occur either in psychiatric or neurological disorders. In this regard, it is important to mention that previous studies evinced the role of Rhes in psychiatric disorders [42,43,44], and described the presence of neuroinflammatory processes in these disorders [11,12,13]. Regarding the role of Rhes in psychiatric disorders, the work of Liu and colleagues [42] demonstrated that Rhes may be a potential vulnerability factor for schizophrenia, whereas Rhes was also found to influence schizophrenia-related transduction pathways within the STR, such as of Akt and mTOR [43,44]. Moreover, microglia activation has been found in major depressive, bipolar and schizophrenic disorders [11,12,13]. 

In addition to what described in psychiatric disorders, microglial activation and accumulation of proinflammatory cytokines are associated with degeneration of DAergic neurons in SNc of patients with PD [45,46,47], as well as in various PD animal models [11,12,13,28,39,48,49]. 

All together, these results underline the important role of Rhes in pathologies that involve the DAergic system.

Results concerning DA neuron degeneration evaluated through TH immunohistochemistry demonstrated that both adult WT and Rhes KO mice were affected by MDMA administration, although male mice showed a more marked decrease in TH immunoreactivity in both STR and SNc. Importantly, the decrease in TH protein levels was not only functional, since previous studies, using Nissl staining, showed that the protocol of MDMA applied in this study is able to induce neuronal loss in this area [31]. Moreover, evaluation of the relationship between this decrease and gender revealed a further important result by showing that adult male Rhes KO mice showed a marked decrease, especially in STR and SNc, whereas female Rhes KO mice did not. This result is in line with our previous paper which reported that in basal conditions, adult male Rhes KO mice displayed a significant decrease in TH-positive fibres in STR compared with WT mice, whereas no modifications were observed in adult female Rhes KO mice compared with WT mice [10].

Regarding middle-aged mice, a significant decrease of TH-positive fibres and neurons was observed both in male and female WT and Rhes KO mice, although, again in males, the decrease was more marked than in females. MDMA administration showed a DAergic neurodegenerative effect in STR and SNc of adult male WT and Rhes KO mice, both adults and middle-aged, whereas in female Rhes KO, the effect was age-dependent.

Collectively, our data give support to the influence of Rhes in regulating the survival of DA neurons [7]. The present results are also in line with previous findings showing that MDMA may induce neuroinflammatory and toxic effects in a gender-dependent manner and that males are more susceptible to these effects [50,51,52]. Although the results obtained in the present study confirm that Rhes signaling pathways are implicated in glial activation and neuronal survival [6,10,51,52,53,54], the mechanism that forms the basis of glial activation and DA neuron degeneration in Rhes KO mice must be elucidated by further studies.

The results on gliosis and DA neuron degeneration, by disclosing a higher susceptibility of male Rhes KO than females, highlight the different response of the two genders to neuroinflammatory and neurotoxic amphetamine-related drugs effects, posing a further concern regarding the use of these substances as recreational drugs.

Previous studies have shown that lack of Rasd2 (Rhes) in KO mice enhances the behavioural sensitivity to motor stimulation elicited by amphetamine and modulates sensorimotor gating in a gender-dependent manner [2,5,6,55], suggesting again that Rhes might represent a potential vulnerability factor for neurological and psychiatric disorders.

Therefore, in light of these previous findings, we additionally evaluated the motor response of adult male and female WT and Rhes KO mice to MDMA. Results confirmed that, as for other amphetamine-related drugs, Rhes KO male mice display a higher motor stimulant effect in response to MDMA than WT controls. In addition, we observed that the motor response observed in male Rhes KO mice is higher than in female Rhes KO male mice, on both locomotor and total (locomotor plus stereotypies) motor activity, again suggesting a differential response to MDMA between male and female Rhes KO mice. This result might appear in contrast with the decrease in TH-positive neurons observed. However, since TH is only partially decreased, this effect may produce a compensation at postsynaptic receptor level that become more sensitive to the DA released by MDMA from the DAergic remaining terminals, increasing the motor response induced by the drug. Our findings are consistent with data reported by Shahani et al.; which demonstrate that partially Rhes-deficient Rhes^+/−^ mice had an enhanced locomotor response to amphetamine, although that report did not show the differential effect induced by amphetamine in male and female mice [55]. Moreover, our results are in line with the finding showing that apomorphine, a DA D_1_/D_2_ receptor agonist, induced higher levels of stereotypies in male Rhes KO mice than in female Rhes KO mice [56]. It should be noted, however, as reported by Ghiglieri et al.; that the locomotor activity induced by the DA D_1_ agonist SKF 81297 is higher in female Rhes KO mice than in male Rhes KO mice, suggesting a different response of DA D_1_ receptors in Rhes KO mice [3]. 

In our previous papers, we have suggested that Rhes KO mice might represent a model in which to study glial activation and DA neuron degeneration associated to psychiatric and neurological disorders such as PD [7,10]. The result of a higher vulnerability of neuroinflammation and DA neuron degeneration by MDMA in Rhes KO male mice than in females, and the dependence from age, add further interest in the role of the Rhes protein in neuropsychiatric and neurodegenerative diseases.

## 4. Materials and Methods 

### 4.1. Drugs

MDMA HCl was synthesised by Prof. Antonio Plumitallo at the Department of Life and Environmental Sciences, University of Cagliari, as described elsewhere [34] and was dissolved in saline. The dose of MDMA used, which is compatible with an effective dose translated to mice, was chosen on the basis of previous studies [31,38] and may be regarded as medium/high dose, considering studies performed in humans [57,58].

### 4.2. Animals and Treatments

Adult (3-month-old) and middle-aged (12-month-old) male and female WT and Rhes KO mice were used in this study and derived from mating of heterozygous mice (Rhes^+/−^) back-crossed to F11 generation to C57BL/6 strain, were treated with vehicle (saline solution) or MDMA (4 × 20 mg/kg intraperitoneally (i.p.), 2-intervals). Mice were housed in groups of four to six in standard polycarbonate cages with sawdust bedding and maintained on a 12-h/12-h light/dark cycle (lights on at 8:00 am). Food and water were freely available, except during the motor activity measurement, which was performed between 12:00 and 4:00 pm. All experiments were conducted in accordance with guidelines for animal experimentation (D.L. no. 26 on 4 March 2014, Implementation of the Directive 2010/63/EU on the protection of animals used for scientific purposes), with the license issued by the Italian Ministry of health (529/2016-PR, released on 14 March 2016) and with guidelines approved by the ethics committee of the University of Cagliari. Experiments were designed to minimise animal discomfort as much as possible and to reduce the number of animals used.

### 4.3. Immunohistochemistry

Immunohistochemical evaluation was performed in a group of mice different from that used for motor activity. Animals were euthanised 48 h after the last administration of MDMA in order to have the greatest microglial activation [31,39,59,60].

### 4.4. Analysis of GFAP Immunoreactivity

Images were digitised (Axio Scope.A1; Carl Zeiss Microscopy, Oberkochen, Germany) under constant light conditions. Sections were captured at 20× magnification (for CTX and STR analysis) or at 10× magnification (for NAc, SNc and VTA analysis). Analysis was performed in a blinded manner in the three sections. The number of GFAP-positive cells was determined quantitatively in (i) one portion from the M1 and one portion from the M2 CTX, (ii) the dorsolateral and ventromedial STR, (iii) two portions of the NAc, (iv) the whole SNc; (v) the whole VTA in all the left and right hemispheres of the brain areas evaluated, using the Multi-point Tool of the Image J software program (National Institutes of Health, Bethesda, MD, USA). Astroglial cells were counted when a cell body from which processes extended was observed or when the processes were all directed toward a central point that corresponded with the likely position of the cell body deeper in the tissue. GFAP-expressing fibres without a clear indication of the associated cell body were not counted. For each level of the CTX, STR; NAc, SNc or VTA, the obtained values from the different levels were averaged.

### 4.5. Analysis of CD11b Immunoreactivity

Images were digitised in greyscale and captured at 10× magnification (for STR, NAc and VTA analysis) or at 20× magnification (for CTX and SNc analysis). Analysis was performed in a blinded manner in the three sections. The levels of CD11b were determined quantitatively in (i) one portion from the M1 and one portion from the M2 CTX, (ii) the dorsolateral and ventromedial STR, (iii) two portions of the NAc, (iv) the whole SNc; (v) the whole VTA in all the left and right hemispheres of the brain areas evaluated, using the Image J program (National Institutes of Health, Bethesda, MD, USA). Before starting the analysis, in the “Set measurements” Image J dialog box, we selected “Mean grey values”, then the area of interest was selected for each image using the Polygon Selection Tool. In this manner we were able to obtain the average grey value within the selection. This value is the sum of the grey values of all the pixels in the selection divided by the number of pixels. For each level of the CTX, STR, NAc, SNc or VTA the obtained values from different levels were averaged.

### 4.6. Analysis of TH Immunoreactivity in the CTX, STR or NAc

Images were digitised (Axio Scope.A1) in greyscale and captured at 5× (for STR), 10× (for NAc), 20× (for CTX) magnification. Analysis was performed in a blinded manner in the three sections. The density of immunoreacted fibres was determined quantitatively using Image J. No significant differences in the density of immunoreacted fibres were seen between the three sections, thus values from different levels were averaged.

### 4.7. Stereological Counting of TH-Immunoreactive Neurons in the SNc or VTA

Stereological analysis of total number and density of TH-positive neurons in the SNc or VTA was done in both hemispheres using a software (Stereologer; SRC Biosciences, Tampa, FL, USA) linked to a motorised stage on a light microscope [61]. The SNc or the VTA regions were outlined at low magnification (2×), and sampling of cells was achieved using automatically randomised sampling and an optical dissector (50 × 50 × 15 µm). Cells were sampled with a 40× objective through a defined depth with a guard zone of 2 µm. Coefficient of error ranged from 0.05 to 0.1 [61].

### 4.8. Motor Activity Measurement

Measurement of motor activity was carried out in a quiet, isolated room. Each mouse was placed individually in a cage (length, 47 cm; width, 27 cm; height, 19 cm) equipped with a horizontal infrared beam emitter-detector system (Opto-Varimex; Columbus Instruments, Columbus, OH, USA). The interruption of a photocell beam was detected by a counter that recorded the total number of photocell beam interruptions. The counter recorded two different types of motor activity: (1) locomotor activity due to the locomotion of the mouse along the axes of the cage and (2) total motor activity due to locomotion plus non finalised movements (stereotyped behaviours such as grooming, rearing and sniffing). The counter recognised the stereotyped movements because of the continuous interruption of the same photocell beam, whereas locomotion along the cage produced interruptions of different photocell beams. Mice were habituated to the cages for 1 h before the first vehicle or MDMA administration. Activity counts were taken every 15 min, for a total of four evaluations (cumulative time, 1 h), starting after each injection. Pharmacological treatments and motor activity measurement were performed in a room kept at a constant temperature of 21 ± 1 °C.

### 4.9. Statistics

Statistical analysis was performed with Statistica for Windows software (version 8, StatSoft, Tulsa, OK, USA). Data from the immunohistochemical analysis were statistically compared by means of a four-way (gender × genotype × age × treatment) analysis of variance (ANOVA). ANOVA was followed by Tukey’s post hoc test. Data from motor activity measurements were analysed with repeated measures ANOVA (gender × genotype × treatment × time), followed by the Tukey’s post hoc test. Results were considered significant at *p* < 0.05, and the results are expressed as mean ± SEM for every analysis performed. 

## Figures and Tables

**Table 1:** Immunoreactivity for the glial fibrillary acidic protein (GFAP) in the motor cortex (CTX), nucleus accumbens (NAc), and ventral tegmental area (VTA).

GFAP					
		Adult Male	Adult Female	Middle-Aged Male	Middle-Aged Female
VTA	Rhes^+/+^ Vehicle	41.31 ± 3.06	28.25 ± 1.18	32.12 ± 1.48	28.62 ± 0.80
	Rhes^+/+^ MDMA	42.62 ± 5.28	28.63 ± 1.19	38.12 ± 1.93	30.56 ± 2.03
	Rhes^−/−^ Vehicle	43.44 ± 1.25	31.19 ± 1.57	32.75 ± 1.46	31.56 ± 2.13
	Rhes^−/−^ MDMA	46.62 ± 3.44 ^^^	30.06 ± 2.66	39.44 ± 2.32	30.62 ± 2.08
NAc	Rhes^+/+^ Vehicle	5.37 ± 0.55	3.92 ± 0.35	4.08 ± 0.26	3.79 ± 0.34
	Rhes^+/+^ MDMA	4.96 ± 0.48	4.08 ± 0.25	4.62 ± 0.26	3.83 ± 0.43
	Rhes^−/−^ Vehicle	4.50 ± 0.26	4.54 ± 0.51	5.08 ± 0.26	3.71 ± 0.38
	Rhes^−/−^ MDMA	4.60 ± 0.65	4.52 ± 0.33	4.96 ± 0.42	3.37 ± 0.16
CTX	Rhes^+/+^ Vehicle	29.46 ± 0.99	25.73 ± 3.61	39 ± 1.61	30.04 ± 1.01
	Rhes^+/+^ MDMA	33.33 ± 0.67	26.13 ± 1.29	48.62 ± 2.22	31.87 ± 0.31
	Rhes^−/−^ Vehicle	30.29 ± 3.43	21.67 ± 0.83	37.54 ± 2.22	31.37 ± 1.86
	Rhes^−/−^ MDMA	35.87 ± 3.13	21.69 ± 1.53	47.92 ± 5.32	32.37 ± 1.90

Representative values of the CTX, NAC and VTA immunostained for GFAP of adult and middle-aged male and female WT and Rhes KO mice treated with vehicle or MDMA (4 × 20 mg/kg i.p.; 2-h intervals). The total number of GFAP-positive cells are expressed as mean ± S.E.M. Number of mice per group: vehicle-treated WT adult male: *n* = 8 and female: *n* = 8; MDMA-treated WT adult male: *n* = 8 and female: *n* = 9; vehicle-treated WT middle-aged male: *n* = 12 and female: *n* = 6; MDMA-treated WT middle-aged male: *n* = 7 and female: *n* = 6; vehicle-treated Rhes KO adult male: *n* = 8 and female: *n* = 9; MDMA-treated Rhes KO adult male: *n* = 8 and female: *n* = 10; vehicle-treated Rhes KO middle-aged male: *n* = 8 and female: *n* = 6; MDMA-treated Rhes KO middle-aged male: *n* = 6 and female: *n* = 5. ^^^ *p* < 0.05 compared with the age- and genotype-matched female mice by Tukey post hoc test.

**Table 2:** Immunoreactivity for complement type 3 receptor (CD11b) in the motor cortex (CTX), nucleus accumbens (NAc), and ventral tegmental area (VTA).

CD11b					
		Adult Male	Adult Female	Middle-Aged Male	Middle-Aged Female
VTA	Rhes^+/+^ Vehicle	43.25 ± 0.46	46.19 ± 1.71	47 ± 0.46	45.5 ± 1.57
	Rhes^+/+^ MDMA	51.56 ± 0.46 *	50.63 ± 0.78	54.94 ± 0.92 *	53.31 ± 0.97 *
	Rhes^−/−^ Vehicle	49.37 ± 1.29	49.19 ± 0.22	50.94 ± 0.26	49.5 ± 0.57
	Rhes^−/−^ MDMA	56.44 ± 1.17 *	53.19 ± 0.28	60.5 ± 1.09 **	58.25 ± 1.75 **
NAc	Rhes^+/+^ Vehicle	47.33 ± 1.94	50.06 ± 2.95	65.62 ± 1.20 ^$$^	58.58 ± 1.96
	Rhes^+/+^ MDMA	63.66 ± 1.86 *	59.25 ± 0.49	73.87 ± 2.99	67.42 ± 0.78
	Rhes^−/−^ Vehicle	52.64 ± 0.58	57.42 ± 1.45	66.92 ± 0.79 ^$^	58.13 ± 1.85
	Rhes^−/−^ MDMA	62.12 ± 1.07	59.66 ± 0.73	78 ± 5.73 ^$^	66.42 ± 0.33
CTX	Rhes^+/+^ Vehicle	50.66 ± 0.66	54.29 ± 0.25	54.12 ± 0.85	50.54 ± 0.41
	Rhes^+/+^ MDMA	56.16 ± 0.77 *	61.69 ± 0.43 **	63.29 ± 0.89 ***^$$^	56.92 ± 0.35 **
	Rhes^−/−^ Vehicle	51.12 ± 1.50	54.21 ± 1.05	61.25 ± 1.27 ^##$$$^^^^	53.12 ± 0.96
	Rhes^−/−^ MDMA	57.75 ± 0.70 *	59.67 ± 0.37 *	69.33 ± 0.50 ^***#$$$^^^^	58.29 ± 0.18

Representative values of the CTX, NAC and VTA immunostained for CD11b of adult and middle-aged male and female WT and Rhes KO mice treated with vehicle or MDMA (4 × 20 mg/kg i.p.; 2-h intervals). The mean density of grey values are expressed as mean ± S.E.M. Number of mice per group: vehicle-treated WT adult male: *n* = 8 and female: *n* = 8; MDMA-treated WT adult male: *n* = 8 and female: *n* = 9; vehicle-treated WT middle-aged male: *n* = 12 and female: *n* = 6; MDMA-treated WT middle-aged male: *n* = 7 and female: *n* = 6; vehicle-treated Rhes KO adult male: *n* = 8 and female: *n* = 9; MDMA-treated Rhes KO adult male: *n* = 8 and female: *n* = 10; vehicle-treated Rhes KO middle-aged male: *n* = 8 and female: *n* = 6; MDMA-treated Rhes KO middle-aged male: *n* = 6 and female: *n* = 5. * *p* < 0.05, ** *p* < 0.005and *** *p* < 0.0005 compared with the respective vehicle; ^#^ *p* < 0.05 and ^##^ *p* < 0.005 compared with the age- and gender-matched WT mice; ^^^^^ *p* < 0.0005 compared with the age- and genotype-matched female mice; ^$^ *p* <0.05, ^$$^ *p* <0.005 and ^$$$^ *p* < 0.0005 compared with the respective adult mice by Tukey post hoc test.

**Table 3:** Immunoreactivity for tyrosine hydroxylase (TH) in the motor cortex (CTX), nucleus accumbens (NAc), and ventral tegmental area (VTA).

TH					
		Adult Male	Adult Female	Middle-Aged Male	Middle-Aged Female
VTA	Rhes^+/+^ Vehicle	128.06 ± 29.15	102.81 ± 16.52	139.25 ± 6.83	91.94 ± 6.23
	Rhes^+/+^ MDMA	141.09 ± 34.01	94.38 ± 25.92	145 ± 10.43	86.13 ± 3.42
	Rhes^-/-^ Vehicle	174.13 ± 44.64	75.81 ± 17.33	151.06 ± 10.48	75.63 ± 5.01
	Rhes^-/-^ MDMA	169.84 ± 41.75	75.81 ± 17.33	153 ± 8.94	75.25 ± 2.36
NAc	Rhes^+/+^ Vehicle	266.01 ± 53.59	209.35 ± 31.81	218.23 ± 0.78	213.96 ± 2.58
	Rhes^+/+^ MDMA	261.96 ± 47.94	201.50 ± 28.37	210.12 ± 2.40	206.39 ± 2.02
	Rhes^-/-^ Vehicle	236.73 ± 38.47	173.80 ± 20.16	218.67 ± 4.16	183.89 ± 7.65
	Rhes^-/-^ MDMA	247.15 ± 42.99	213.33 ± 29.32	206.60 ± 8.46	196.54 ± 4.54
CTX	Rhes^+/+^ Vehicle	33.66 ± 1	25.25 ± 2.13	30.73 ± 1.46	25 ± 0.77
	Rhes^+/+^ MDMA	37.48 ± 0.72	27.71 ± 2.68	31.08 ± 0.64	24.33 ± 0.34
	Rhes^-/-^ Vehicle	35.12 ± 1.51	26.54 ± 1.62	32.83 ± 1.65 ^^^	20.37 ± 1.63
	Rhes^-/-^ MDMA	37.87 ± 1.19	28.75 ± 2.20	31.81 ± 0.50	22.58 ± 0.62

Representative values of the CTX, NAC and VTA immunostained for TH of adult and middle-aged male and female WT and Rhes KO mice treated with vehicle or MDMA (4 × 20 mg/kg i.p.; 2-h intervals). The number of TH-positive neurons in VTA and the mean density of grey values of TH-positive fibres in CTX and NAc are expressed as mean ± S.E.M. Number of mice per group: vehicle-treated WT adult male: *n* = 8 and female: *n* = 8; MDMA-treated WT adult male: *n* = 8 and female: *n* = 9; vehicle-treated WT middle-aged male: *n* = 12 and female: *n* = 6; MDMA-treated WT middle-aged male: *n* = 7 and female: *n* = 6; vehicle-treated Rhes KO adult male: *n* = 8 and female: *n* = 9; MDMA-treated Rhes KO adult male: *n* = 8 and female: *n* = 10; vehicle-treated Rhes KO middle-aged male: *n* = 8 and female: *n* = 6; MDMA-treated Rhes KO middle-aged male: *n* = 6 and female: *n* = 5. ^^^ *p* < 0.05 compared with the age- and genotype-matched female mice by Tukey post hoc test.

## Supplementary Materials

Supplementary materials can be found at https://www.mdpi.com/1422-0067/20/7/1556/s1.

## Abbreviations

A	anteriority
ABC	avidin–biotin–peroxidase complex
CD11b	complement type 3 receptor
STR	striatum
CTX	motor cortex
DA	dopamine
DAergic	dopaminergic
GFAP	glial fibrillary acidic protein
KO	knockout
IgG	immunoglobulin G
i.p.	intraperitoneally
NAc	nucleus accumbens
PD	Parkinson’s disease
SNc	substantia nigra pars compacta
TH	tyrosine hydroxylase
VTA	ventral tegmental area
WT	wild-type
