# Supplementary material for: Lack of Rhes Increases MDMA-Induced Neuroinflammation and Dopamine Neuron Degeneration: Role of Gender and Age
Source: Int J Mol Sci. 2019 Mar 28;20(7):1556. doi: 10.3390/ijms20071556 (PMC6480667; doi:10.3390/ijms20071556)
Supplement: Supplementary file 1 [file ijms-20-01556-s001.pdf]

Supplementary Statistic

Effects of MDMA on GFAP immunoreactivity

Four-way ANOVA of GFAP immunoreactivity in the CTX revealed a significant effect of treatment ($F_{1,48}$ = 6.64, p<0.05) and a significant gender × treatment interaction ($F_{1,48}$ = 4.26, p<0.05). Moreover, in the CPu a significant effect of gender ($F_{1,95}$ = 12.75, p<0.001), genotype ($F_{1,95}$ = 6.55, p<0.05) and treatment ($F_{1,95}$ = 358.44, p<0.0001) was observed. Furthermore, in the NAc a significant effect of gender ($F_{1,48}$ = 8.97, p<0.005) and a significant gender × genotype × age interaction ($F_{1,48}$ = 5.18, p<0.05) was observed. Finally, in the SNc, a significance of gender ($F_{1,97}$ = 17.22, p<0.001), genotype ($F_{1,97}$ = 11.60, p<0.001), age ($F_{1,97}$ = 30.34, p<0.0001), treatment ($F_{1,97}$ = 22.65, p<0.001) and a significant gender × genotype ($F_{1,97}$ = 8.50, p<0.005) and gender × age ($F_{1,97}$ = 96.79, p<0.0001) interactions were also observed, whereas in VTA a significant effect of gender ($F_{1,48}$ = 42.81, p<0.0005), a significant effect of age ($F_{1,48}$ = 5.86, p<0.05) and a significant gender × age interaction ($F_{1,48}$ = 8.59, p<0.05) was observed.

Effects of MDMA on CD11b immunoreactivity

Four-way ANOVA of CD11b immunoreactivity in the CTX revealed a significant effect of gender ($F_{1,48}$ = 13.60, p<0.005), genotype ($F_{1,48}$ = 17.47, p<0.0005), age ($F_{1,48}$ = 26.66, p<0.0005), treatment ($F_{1,48}$ = 169.81, p<0.0005), and significant gender × genotype ($F_{1,48}$ = 8.70, p<0.005), gender × age ($F_{1,48}$ = 111.78, p<0.0005), genotype × age ($F_{1,48}$ = 17.47, p<0.005) interactions. Moreover, in the CPu a significant effect of gender ($F_{1,90}$ = 33.24, p<0.0001), genotype ($F_{1,90}$ = 24.78, p<0.001), treatment ($F_{1,90}$ = 50.68, p<0.0001), and a significant gender × genotype ($F_{1,90}$ = 7.195, p<0.01), age × treatment ($F_{1,90}$ = 5.51, p<0.05) and gender × genotype × age ($F_{1,90}$ = 18.10, p<0.001) interactions were observed. Furthermore, in the NAc a significant effect of gender ($F_{1,48}$ = 9.41, p<0.005), age ($F_{1,48}$ = 60.66, p<0.0005), treatment ($F_{1,48}$ = 47.49, p<0.0005), and a significant gender × age interaction ($F_{1,48}$ = 11.23, p<0.005) was observed. Finally, in the SNc a significance of gender ($F_{1,94}$ = 30.55, p<0.0001), genotype ($F_{1,94}$ = 9.98, p<0.005), treatment ($F_{1,94}$ = 80.58,

$p<0.0001$), and gender × treatment ($F_{1,94} = 13.36$, $p<0.001$) and gender × genotype × age ($F_{1,94} = 5.58$, $p<0.05$) interactions were observed, whereas in VTA a significant effect of genotype ($F_{1,48} = 40.95$, $p<0.0005$), age ($F_{1,48} = 12.94$, $p<0.005$) and treatment ($F_{1,48} = 116.42$, $p<0.0005$) were observed.

Effects of MDMA on TH immunoreactivity

Four-way ANOVA of TH immunoreactivity in the CTX revealed a significant effect of gender ($F_{1,48} = 73.61$, $p<0.0005$) and age ($F_{1,48} = 16.27$, $p<0.0005$). Moreover, in CPu a significant effect of gender ($F_{1,96} = 112.47$, $p<0.0001$), genotype ($F_{1,96} = 57.80$, $p<0.0001$), age ($F_{1,96} = 259.84$, $p<0.0001$), treatment ($F_{1,96} = 69.36$, $p<0.0001$), and a significant gender × genotype ($F_{1,96} = 24.35$, $p<0.001$), gender × age ($F_{1,96} = 37.21$, $p<0.0001$) and gender × treatment ($F_{1,96} = 19$, $p<0.001$) interactions were also observed in CPu. Furthermore, in the NAc a significant effect of gender ($F_{1,48} = 17.34$, $p<0.0005$) and age ($F_{1,48} = 292.17$, $p<0.0005$) was observed. Finally, in SNc a significant effect of gender ($F_{1,108} = 67.67$, $p<0.0001$), genotype ($F_{1,108} = 51.96$, $p<0.0001$), age ($F_{1,108} = 71.94$, $p<0.0001$) and treatment ($F_{1,108} = 77.70$, $p<0.0001$), and a significant gender × age ($F_{1,108} = 144.47$, $p<0.0001$), gender × treatment ($F_{1,108} = 8.41$, $p<0.005$), genotype × treatment ($F_{1,108} = 5.79$, $p<0.05$), age × treatment ($F_{1,108} = 7.75$, $p<0.01$) interactions were observed, whereas in VTA a significant effect of gender ($F_{1,60} = 10.65$, $p<0.005$) and age ($F_{1,60} = 45.16$, $p<0.0005$) and a significant gender × age interaction ($F_{1,60} = 56.98$, $p<0.0005$) was observed.
